# The impact of demographic change on value set validity and obsolescence

**DOI:** 10.1007/s11136-024-03770-5

**Published:** 2024-09-13

**Authors:** Marcel F. Jonker

**Affiliations:** 1 Erasmus School of Health Policy & Management, Erasmus University Rotterdam, Rotterdam, The Netherlands; 2 Erasmus Choice Modelling Centre, Erasmus University Rotterdam, Rotterdam, The Netherlands

**Keywords:** Value set validity, Value set redundancy, Value set obsolescence, Demographic trends, EQ-5D

## Abstract

**Purpose:**

To investigate the contribution of demographic trends in countries’ age and gender composition to value set validity and obsolescence.

**Methods:**

Time-trade off (TTO) valuation data from 3 EQ-5D-3L value sets of 20 years or older from the United Kingdom, Japan, and the United States were re-analyzed using Bayesian heteroskedastic Tobit models with sex and age group-specific scale parameters. Original value sets were obtained by weighting the original preference structures with the countries’ original demographic composition at the time of the data collection. Updated value sets were created using the original preference structure weighted using the countries’ most recent demographic composition. The differences between the original and updated value sets were monitored and compared based on 95% credible intervals.

**Results:**

The gender and age composition of the investigated countries changed in all 3 countries over time. The modelled health state preferences also depended on the respondents’ gender and age. However, the overall impact of this demographic change on the investigated value sets was negligeable in all 3 countries and this finding was robust to accounting for the impact of ethnicity trends in the United States.

**Conclusion:**

Value sets may become redundant and obsolete for various reasons, but demographic change was not identified as a contributing factor.

**Supplementary Information:**

The online version contains supplementary material available at 10.1007/s11136-024-03770-5.

## Introduction

The use of preference-based value sets in health technology assessment (HTA) and other applications is widespread, and the validity of reported results depends on the value sets reflecting contemporary societal preferences [1]. On the one hand, decision-makers often prioritize consistency in value sets, at times prescribing specific sets for analyses to maintain consistency in between-study comparisons [1, 2]. Consequently, they may hesitate to abandon older value sets. On the other hand, several factors can render value sets increasingly outdated and eventually obsolete over time. These factors include the development of improved methods for eliciting and modelling preferences, changes in populations’ preferences, and shifts in the demographic composition of populations over time.

To date, there are no universally accepted expiration dates or guidelines for reassessing the validity of value sets. In fact, the concept of value set invalidity and subsequent obsolescence has received, with some notable exceptions, very little attention in the literature [3, 4]. At the same time, value sets are becoming older and are seldom updated. Hence there is a growing need to investigate the determinants of value set obsolescence. This paper focuses on a specific aspect of value set obsolescence, which is the impact of demographic shifts in the demographic composition of populations. More specifically, it aims to determine whether changes in the age/sex composition of populations, all other things being equal (ceteris paribus), can implicitly generate an expiration date for preference-based value sets.

## Methods

To evaluate the impact of demographic change on preference-based values sets, 3 of the oldest EQ-5D value sets were selected for re-analysis: those of the United Kingdom (UK) [5, 6], Japan (JPN) [7], and the United States (US) [8]. Permission to re-analyze the data was obtained from the corresponding authors and the time trade-off (TTO) valuation data were subsequently obtained from public data repositories and from the corresponding author of the Japanese value set. The obtained data comprised, in addition to the TTO-derived health-state values, also the participating respondents’ sex and age, and in the case of the USA, the respondents’ race/ethnicity, which was used in a sensitivity analysis that is reported in the online supplemental materials. The respondents’ age was recoded into 4 groups, i.e. 18–34, 35–54, 55–74, and 75+.

To allow for an evaluation of the impact of changes in the age and sex composition of the populations of these 3 countries, demographic data were obtained from the World Bank [9]. Both the demographic composition in the year the TTO data were collected and the most recent demographic composition from the year 2022 was obtained. This allowed the original value sets to be obtained by weighting the estimated preference structures with the countries’ original demographic composition at the time of the data collection, and updated value sets by weighting the original preference structure with the countries’ most recent demographic composition.

This was performed by re-analyzing the TTO valuation using state-of-the-art methods [10]. In the Japanese dataset, each respondent completed the same set of 17 TTO tasks. In the UK and US datasets, each respondent completed 12 TTO tasks that were included in the analysis, which were sampled from an overall design that comprised 45 EQ-5D-3 L health states. First, respondents with fewer than 10 observations were excluded from the analyses. Second, respondents with a positively sloped relationship between their TTO valuations and the misery index of the health states were excluded from the analyses. Third, the data of the remaining respondents was analyzed using Bayesian heteroskedastic Tobit models with censoring at -1 using a model specification that included demographic-specific scale parameters. More specifically, the observed TTO values for respondent i in task t were censored as:

1 $$\:{\text{T}\text{T}\text{O}}_{\text{i}\text{t}}=\left\{\begin{array}{ll}{{\:TTO}_{it}^{*}}&{if\quad{TTO}_{it}^{*}>-1}\\{-1}&{if \quad{TTO}_{it}^{*}\le\:-1}\end{array}\right.$$

with the latent $\:{TTO}_{it}^{*}$ values assumed to be normally distributed:

2 $$\:{\text{T}\text{T}\text{O}}_{it}^{*}\sim Normal\left(\:{\mu\:}_{it},{\sigma\:}_{it}\right).$$

Here the mean $({\mu\:}_{it})$ and standard deviation $(\:{\sigma\:}_{it})$ reflect the average health state value and variation among respondents in their valuation of the health state presented in task t of respondent i, respectively. Similar to Pickard et al. [11]., the standard deviation $(\:{\sigma\:}_{it})$ was modelled as a 4th-order polynomial of the health state values:

3 $$\:{{\sigma\:}_{it}=exp(\:{{\gamma\:}}_{0}+{{\gamma\:}}_{1}\mu\:}_{\text{i}\text{t}}+{{\gamma\:}}_{2}{\mu\:}_{it}^{2}+{{\gamma\:}}_{3}{\mu\:}_{it}^{3}+{{\gamma\:}}_{4}{\mu\:}_{it}^{4})$$,

which ensured that the variances of the predicted values could flexibly depend on the health state severity.

The mean$(\:{\mu\:}_{it})$ was specified as follows:

4 $$\begin{aligned}{{\mu\:}}_{\text{i}\text{t}}&={\beta\:}_{0}+\left({\beta\:}_{1}{\text{MO2}}_{it}+{\beta}_{2}{\text{MO3}}_{it}\right)*{{\varphi\:}\_\text{M}\text{O}}_{sa}\\&\;+\left({\beta\:}_{3}{\text{SC2}}_{it}+{\beta\:}_{4}{\text{SC3}}_{it}\right)*{{\varphi\:}\_\text{S}\text{C}}_{sa}\\&\;+({\beta\:}_{5}{\text{UA2}}_{it}+{{\:\beta\:}_{6}\text{UA3}}_{it}){\text{*}{\varphi\:}\_\text{U}\text{A}}_{sa}\\&\;+{\beta\:}_{7}{PD2}_{it}{+\:\beta\:}_{8}{PD3}_{it})*{{\varphi\:}\_\text{P}\text{D}}_{sa}\\&\;+\left({\beta\:}_{9}{AD2}_{it}{+\:\beta\:}_{10}{AD3}_{it}\right)*\:{{\varphi\:}\_\text{A}\text{D}}_{sa}.\end{aligned}$$

Here $\:{\beta\:}_{0}$ denotes the intercept, $\:{\beta\:}_{1-10}$ denote the slope coefficients that capture the EQ-5D decrements for levels 2 and 3, and the $\:\varphi\:$ parameters are multiplicative scale parameters that shrink or amplify respondents’ slope coefficients based on their sex s and age group a $(a\:\in\:\left\{1-4\right\})$, which amounts to 8 scale parameters $(\:\varphi\:)$ per EQ-5D dimension. For statistical identification, the scale parameters were constrained to be positive and subject to a mean-of-one constraint.

Bayesian Markov Chain Monte Carlo (MCMC) methods were used to fit the models, which involves the selection of prior distributions for the unknown parameters and updating these via the likelihood of the observed data. Uninformative normal priors with a mean of zero and standard deviation of 10 were assigned to the $\:\beta\:$ and $\:\gamma\:$ parameters and mean-to-one constrained log-normal parameters were assigned to the dimension-specific $\:{\varphi\:}$ parameters. Similar to Jonker et al. [12], the standard deviation of the log-normal priors was set to 0.4 and the mean of the log-normal priors was defined as µ = -σ^2^/2 = -0.08 to ensure that the prior distribution had an expectation of 1. The models were implemented in the BUGS language and fitted using OpenBUGS [13]. A custom-implemented Metropolis-within-Gibbs algorithm with antithetic sampling was used to update the $\:\beta\:$ and $\:\gamma\:$ parameters and a custom-implemented slice sampling algorithm was used to update the dimension-specific $\:{\varphi\:}$ parameters subject to the mean-of-one constraints [12]. All reported estimates were based on 10,000 burn-in iterations to let three chains converge and a total of 30,000 MCMC iterations to reliably approximate the posterior distributions. Note that the model codes are included in the online supplemental and that convergence was evaluated based on a visual inspection of the MCMC chains and the convergence diagnostics as implemented in the OpenBUGS package.

During the Bayesian MCMC estimation, the original value sets were monitored by multiplying the $\:\beta\:$ parameters with the corresponding $\:{\varphi\:}$ scale parameters and then taking a weighted average using weights determined as the proportion of the population in the respective sex and age categories of the scale parameters at the time of the TTO data collection. Similarly, updated value sets were monitored by taking a weighted average of the original preference structure with weights determined by the countries’ most recent demographic composition. Finally, also the difference between the original and updated value sets was monitored during the model estimations. This allowed for a direct assessment of the impact of the change in countries’ age and gender compositions on the value sets.

## Results

The UK valuation dataset contained 3,395 respondents, 82 respondents who completed fewer than 10 TTO observations and 43 respondents with a positively sloped relationship between their TTO valuations and the misery indices of the health states were excluded, resulting in 3,270 respondents. The Japanese valuation dataset contained 543 respondents, 3 respondents who completed fewer than 10 TTO observations and 4 respondents with positive slopes were excluded, resulting in 536 respondents. And the United States valuation dataset contained 4,048 respondents, all of whom completed 10 or more TTO tasks and 133 respondents with positive slopes were excluded, resulting in 3,915 respondents.

Table 1 presents the demographic composition of the samples and compares them to the nationally representative benchmarks. None of the samples were accurately nationally representative at the time of the original data collection. This underscores the need to obtain regression-weighted value sets, both using the original and updated demographic weights. Also, in all three countries the population compositions have shifted and became significantly older in the last 2 decades; most prominently in Japan but also in the other 2 countries.

**Table 1:** Survey respondents and national representative benchmarks, by country, sex, age group, and year

		Country													
		United Kingdom					Japan					United States			
		Dataset	National population				Dataset	National population				Dataset	National population		
Sex	Age	(*N* = 3,270)	1997	2022	Δ		(*N* = 536)	1998	2022	Δ		(*N* = 3,915)	2002	2022	Δ
Male	18–34	13.9%	15.6%	13.8%	-1.8%		9.7%	15.2%	9.9%	-5.3%		16.1%	16.2%	14.9%	-1.3%
Male	35–54	13.9%	17.5%	16.0%	-1.5%		15.5%	17.2%	15.9%	-1.3%		16.5%	19.4%	16.3%	-3.1%
Male	55–74	12.5%	11.5%	14.0%	2.5%		15.5%	13.3%	14.8%	1.5%		7.5%	10.1%	13.9%	3.8%
Male	75+	3.1%	3.3%	5.1%	1.8%		1.3%	2.9%	7.6%	4.7%		2.0%	2.9%	3.9%	1.0%
Female	18–34	18.1%	15.7%	13.3%	-2.4%		12.7%	14.5%	9.5%	-5.0%		22.3%	15.7%	14.5%	-1.2%
Female	35–54	16.9%	17.7%	16.4%	-1.3%		25.6%	16.9%	15.4%	-1.5%		22.2%	19.9%	16.2%	-3.7%
Female	55–74	16.0%	12.7%	14.8%	2.1%		17.7%	14.7%	15.5%	0.8%		10.0%	11.0%	15.1%	4.1%
Female	75+	5.6%	6.0%	6.6%	0.6%		2.1%	5.3%	11.4%	6.1%		3.3%	4.8%	5.2%	0.4%
Total	18+	100%	100%	100%			100%	100%	100%			100%	100%	100%	

The Tobit regression coefficients are presented in the online supplemental. Briefly summarized, in all 3 countries all $\:\beta\:$ parameters and 2 or more $\:{\gamma\:}$ parameters are statistically different from zero - in the sense that the 95% credible intervals do not comprise 0. Moreover, in all 3 countries there are sex and age group-specific scale parameters $(\:{\varphi\:})$ that are different from 1 – in the sense that the 95% credible intervals do not comprise 1. In the United Kingdom, all five EQ domains have scale parameters that are significantly different from 1. In the United States, all domains except for mobility are significantly different from 1, and in Japan, the pain and discomfort and anxiety and depression domains have scale parameters that are significantly different from 1.

The overall impact of the scale parameters and shifts in demographic compositions (i.e., differential preferences and associated weights) is summarized in Table 2, which provides the original and updated value set decrements as well as their differences. As shown, the value sets themselves are country-specific but the differences between the original and updated value set decrements are close to identical. Most differences are 0.00 or smaller and the maximum difference is 0.01. Most importantly, all differences have 95% credible intervals that comprise 0, implying that the original and updated value sets are not significantly different.

**Table 2:** EQ-5D health state decrements, original and age/sex corrected, by country

	United Kingdom				Japan				United States		
	1997	2022	Δ		1998	2022	Δ		2002	2022	Δ
MO2	-0.06 (-0.07 — -0.06)	-0.06 (-0.07 — -0.06)	0.00 (0.00 — 0.00)		-0.10 (-0.11 — -0.08)	-0.10 (-0.11 — -0.08)	0.00 (0.00 — 0.00)		-0.04 (-0.04 — -0.03)	-0.04 (-0.04 — -0.03)	0.00 (0.00 — 0.00)
MO3	-0.34 (-0.36 — -0.33)	-0.34 (-0.36 — -0.33)	0.00 (0.00 — 0.00)		-0.43 (-0.46 — -0.4)	-0.43 (-0.46 — -0.4)	0.00 (-0.01 — 0.01)		-0.34 (-0.35 — -0.32)	-0.34 (-0.35 — -0.32)	0.00 (0.00 — 0.00)
SC2	-0.11 (-0.12 — -0.11)	-0.12 (-0.12 — -0.11)	0.00 (0.00 — 0.01)		-0.05 (-0.07 — -0.04)	-0.06 (-0.07 — -0.04)	0.00 (0.00 — 0.00)		-0.07 (-0.08 — -0.07)	-0.07 (-0.08 — -0.07)	0.00 (0.00 — 0.00)
SC3	-0.23 (-0.25 — -0.22)	-0.24 (-0.26 — -0.23)	0.01 (0.01 — 0.01)		-0.09 (-0.11 — -0.07)	-0.09 (-0.12 — -0.07)	0.00 (0.00 — 0.01)		-0.24 (-0.25 — -0.23)	-0.24 (-0.26 — -0.23)	0.00 (0.00 — 0.00)
UA2	-0.08 (-0.09 — -0.07)	-0.08 (-0.09 — -0.07)	0.00 (0.00 — 0.00)		-0.03 (-0.05 — -0.02)	-0.03 (-0.05 — -0.02)	0.00 (0.00 — 0.00)		-0.04 (-0.05 — -0.04)	-0.04 (-0.05 — -0.04)	0.00 (0.00 — 0.00)
UA3	-0.22 (-0.23 — -0.21)	-0.22 (-0.23 — -0.21)	0.00 (0.00 — 0.00)		-0.13 (-0.15 — -0.11)	-0.12 (-0.15 — -0.1)	0.00 (-0.01 — 0.00)		-0.17 (-0.18 — -0.16)	-0.18 (-0.19 — -0.16)	0.01 (0.00 — 0.01)
PD2	-0.09 (-0.09 — -0.08)	-0.09 (-0.09 — -0.08)	0.00 (0.00 — 0.00)		-0.06 (-0.07 — -0.05)	-0.06 (-0.07 — -0.04)	0.00 (0.00 — 0.00)		-0.05 (-0.06 — -0.05)	-0.05 (-0.06 — -0.04)	0.00 (0.00 — 0.00)
PD3	-0.47 (-0.48 — -0.45)	-0.47 (-0.48 — -0.45)	0.00 (0.00 — 0.00)		-0.18 (-0.2 — -0.17)	-0.17 (-0.19 — -0.16)	-0.01 (-0.01 — 0.00)		-0.36 (-0.37 — -0.35)	-0.36 (-0.37 — -0.35)	0.00 (0.00 — 0.00)
AD2	-0.12 (-0.13 — -0.11)	-0.12 (-0.13 — -0.11)	0.00 (0.00 — 0.00)		-0.06 (-0.08 — -0.05)	-0.06 (-0.08 — -0.05)	0.00 (0.00 — 0.00)		-0.08 (-0.09 — -0.07)	-0.08 (-0.09 — -0.07)	0.00 (0.00 — 0.00)
AD3	-0.37 (-0.38 — -0.35)	-0.37 (-0.38 — -0.36)	0.00 (0.00 — 0.00)		-0.12 (-0.14 — -0.11)	-0.12 (-0.14 — -0.1)	0.00 (-0.01 — 0.00)		-0.26 (-0.27 — -0.25)	-0.27 (-0.28 — -0.26)	0.00 (0.00 — 0.01)

## Discussion

There are multiple reasons why value sets can become redundant and obsolete over time. For example, valuation methods have significantly improved in the past 2 decades, HTA bodies may require different value sets or methods to be used, and most importantly, population preferences can and likely have shifted over time. In this paper one aspect of value set obsolescence has been investigated, which is the extent to which demographic changes in the age/sex composition of populations have, keeping all other things constant, implicitly created an expiration date on preference-based value sets. Based on the presented results, we can conclude that demographic change is not an important determinant of value set validity.

Strengths of this study are that the results were obtained using the original TTO valuation data from 3 different countries from 3 different continents, a state-of-the-art Bayesian modelling approach, and a conceptually simple yet effective method to obtain both original and updated value sets. A potential weakness is that only age and gender were considered in the presented analyses; for example, the impact of ethnicity was ignored even though it could particularly for the United States have been an important determinant of value set obsolescence. Fortunately, according to the sensitivity analysis presented in the online supplemental, changes in the ethnic composition of the US population over the last two decades did not affect the validity of the US value set. Another potential weakness is that historic demographic trends may be smaller than future changes in populations’ age and sex compositions over time. However, the included countries experienced relatively large demographic shifts in the sex/age composition of their populations [9] and the demographic shifts as experienced over the past 20–25 years are equally large or larger than the predicted shifts in the age/sex distribution in the coming 20–25 years [14]. Accordingly, the preference differentials need to be at least an order of magnitude larger than those presented in this paper before demographic change would be able to significantly contribute to value set obsolescence. In other words, the presented results are robust, although contingent upon the assumption that the original preference structure as observed in the TTO datasets has remained constant over time. Future research is therefore necessary to establish the extent to which health-state preferences have indeed remained constant over time, as well as to determine appropriate criteria and threshold values for value set validity and obsolescence.

## Electronic supplementary material

Below is the link to the electronic supplementary material.

Supplementary Material 1

## Data Availability

The UK valuation data are available at 10.5255/UKDA-SN-3444-1. The US valuation data are available at https://archive.ahrq.gov/professionals/clinicians-providers/resources/rice/EQ5Dproj.html. The Japanese valuation data are not publicly available. The demographic data can be obtained from https://databank.worldbank.org/source/health-nutrition-and-population-statistics.
