# Supplementary material for: The impact of demographic change on value set validity and obsolescence
Source: Qual Life Res. 2024 Sep 13;33(11):3155–60. doi: 10.1007/s11136-024-03770-5 (PMC11541319; doi:10.1007/s11136-024-03770-5)
Supplement: Supplementary file 1 — Supplementary Material 1 [file 11136_2024_3770_MOESM1_ESM.pdf]

“The impact of demographic change on value set validity and obsolescence”

Online Supplemental

OpenBUGS model code

```
model {
  # N = number of respondents
  # n_obs[i] = number of tasks per respondent i
  # weight_old[] and weight_new[] are country-specific weights, loaded as data

  # TTO model
  for (i in 1:N){
   for (t in 1:n_obs[i]){

    # Normal likelihood with censoring at -1
    Y[i,t] ~ dnorm( mu_y[i,t], prec_y[i,t] )C(-1,)

    # Mean and standard deviation of normal distribution
    mu_y[i,t] <- value[i,t]
    prec_y[i,t] <- 1/(sd_y[i,t]*sd_y[i,t])
    sd_y[i,t] <- exp( gamma[1] +
                      gamma[2] * value[i,t] +
                      gamma[3] * value[i,t] * value[i,t] +
                      gamma[4] * value[i,t] * value[i,t] * value[i,t] +
                      gamma[5] * value[i,t] * value[i,t] * value[i,t] * value[i,t] )

    # TTO health state values
    value[i,t] <- beta[11] + (beta[1]  * X[i,t,1] +
                              beta[2]  * X[i,t,2])* scale_MO[agesex_group[i]] +
                             (beta[3]  * X[i,t,3] +
                              beta[4]  * X[i,t,4])* scale_SC[agesex_group[i]] +
                             (beta[5]  * X[i,t,5] +
                              beta[6]  * X[i,t,6])* scale_UA[agesex_group[i]] +
                             (beta[7]  * X[i,t,7] +
                              beta[8]  * X[i,t,8])* scale_PD[agesex_group[i]] +
                             (beta[9]  * X[i,t,9] +
                              beta[10] * X[i,t,10])* scale_AD[agesex_group[i]]
  }}
```

```
# Prior on betas
  beta[1:11] ~ dmnorm(b_zeros[], b_prec[,])
  for (b in 1:11){
   b_zeros[b] <- 0
   for (bb in 1:11){
     b_prec[b,bb] <- equals(b,bb)/100
  }}

  # Prior on gammas
  gamma[1:5] ~ dmnorm(g_zeros[], g_prec[,])
  for (g in 1:5){
   g_zeros[g] <- 0
   for (gg in 1:5){
     g_prec[g,gg] <- equals(g,gg)/100
  }}

  # Prior on scale parameters - subject to mean-of-1 constraints
  # see Jonker et al. (2019) Health Economics for technical details
  scale_MO[1:8] ~ dscale(6.25)
  scale_SC[1:8] ~ dscale(6.25)
  scale_UA[1:8] ~ dscale(6.25)
  scale_PD[1:8] ~ dscale(6.25)
  scale_AD[1:8] ~ dscale(6.25)

  # monitor old (original) value set
  weight_MO_old <- scale_MO[1]*weight_old[1] + scale_MO[2]*weight_old[2] + scale_MO[3]*weight_old[3] + scale_MO[4]*weight_old[4]+
                   scale_MO[5]*weight_old[5] + scale_MO[6]*weight_old[6] + scale_MO[7]*weight_old[7] + scale_MO[8]*weight_old[8]
  weight_SC_old <- scale_SC[1]*weight_old[1] + scale_SC[2]*weight_old[2] + scale_SC[3]*weight_old[3] + scale_SC[4]*weight_old[4]+
                   scale_SC[5]*weight_old[5] + scale_SC[6]*weight_old[6] + scale_SC[7]*weight_old[7] + scale_SC[8]*weight_old[8]
  weight_UA_old <- scale_UA[1]*weight_old[1] + scale_UA[2]*weight_old[2] + scale_UA[3]*weight_old[3] + scale_UA[4]*weight_old[4]+
                   scale_UA[5]*weight_old[5] + scale_UA[6]*weight_old[6] + scale_UA[7]*weight_old[7] + scale_UA[8]*weight_old[8]
  weight_PD_old <- scale_PD[1]*weight_old[1] + scale_PD[2]*weight_old[2] + scale_PD[3]*weight_old[3] + scale_PD[4]*weight_old[4]+
                   scale_PD[5]*weight_old[5] + scale_PD[6]*weight_old[6] + scale_PD[7]*weight_old[7] + scale_PD[8]*weight_old[8]
  weight_AD_old <- scale_AD[1]*weight_old[1] + scale_AD[2]*weight_old[2] + scale_AD[3]*weight_old[3] + scale_AD[4]*weight_old[4]+
                   scale_AD[5]*weight_old[5] + scale_AD[6]*weight_old[6] + scale_AD[7]*weight_old[7] + scale_AD[8]*weight_old[8]
```

```
  value_old[1] <- beta[1] * weight_MO_old
  value_old[2] <- beta[2] * weight_MO_old
  value_old[3] <- beta[3] * weight_SC_old
  value_old[4] <- beta[4] * weight_SC_old
  value_old[5] <- beta[5] * weight_UA_old
  value_old[6] <- beta[6] * weight_UA_old
  value_old[7] <- beta[7] * weight_PD_old
  value_old[8] <- beta[8] * weight_PD_old
  value_old[9] <- beta[9] * weight_AD_old
  value_old[10] <- beta[10] * weight_AD_old


  # monitor new (sex and age-adjusted) value set
  weight_MO_new <- scale_MO[1]*weight_new[1] + scale_MO[2]*weight_new[2] + scale_MO[3]*weight_new[3] + scale_MO[4]*weight_new[4]+
                   scale_MO[5]*weight_new[5] + scale_MO[6]*weight_new[6] + scale_MO[7]*weight_new[7] + scale_MO[8]*weight_new[8]
  weight_SC_new <- scale_SC[1]*weight_new[1] + scale_SC[2]*weight_new[2] + scale_SC[3]*weight_new[3] + scale_SC[4]*weight_new[4]+
                   scale_SC[5]*weight_new[5] + scale_SC[6]*weight_new[6] + scale_SC[7]*weight_new[7] + scale_SC[8]*weight_new[8]
  weight_UA_new <- scale_UA[1]*weight_new[1] + scale_UA[2]*weight_new[2] + scale_UA[3]*weight_new[3] + scale_UA[4]*weight_new[4]+
                   scale_UA[5]*weight_new[5] + scale_UA[6]*weight_new[6] + scale_UA[7]*weight_new[7] + scale_UA[8]*weight_new[8]
  weight_PD_new <- scale_PD[1]*weight_new[1] + scale_PD[2]*weight_new[2] + scale_PD[3]*weight_new[3] + scale_PD[4]*weight_new[4]+
                   scale_PD[5]*weight_new[5] + scale_PD[6]*weight_new[6] + scale_PD[7]*weight_new[7] + scale_PD[8]*weight_new[8]
  weight_AD_new <- scale_AD[1]*weight_new[1] + scale_AD[2]*weight_new[2] + scale_AD[3]*weight_new[3] + scale_AD[4]*weight_new[4]+
                   scale_AD[5]*weight_new[5] + scale_AD[6]*weight_new[6] + scale_AD[7]*weight_new[7] + scale_AD[8]*weight_new[8]

  value_new[1] <- beta[1] * weight_MO_new
  value_new[2] <- beta[2] * weight_MO_new
  value_new[3] <- beta[3] * weight_SC_new
  value_new[4] <- beta[4] * weight_SC_new
  value_new[5] <- beta[5] * weight_UA_new
  value_new[6] <- beta[6] * weight_UA_new
  value_new[7] <- beta[7] * weight_PD_new
  value_new[8] <- beta[8] * weight_PD_new
  value_new[9] <- beta[9] * weight_AD_new
  value_new[10] <- beta[10] * weight_AD_new

  # monitor difference
  for (v in 1:10) { value_diff[v] <- value_old[v] - value_new[v] }
}
```

Table A1. Included and excluded respondents, by dataset, sex, and age group

		country					
		United Kingdom		Japan		United States	
		excluded	included	excluded	included	excluded	included
sex	age	(N=125)	(N=3,270)	(N=7)	(N=536)	(N=133)	(N=3,915)
male	18-34	2.4%	13.9%	14.3%	9.7%	6.8%	16.1%
male	35-54	19.2%	13.9%	14.3%	15.5%	17.3%	16.5%
male	55-74	12.0%	12.5%	28.6%	15.5%	8.3%	7.5%
male	75+	5.6%	3.1%	14.3%	1.3%	3.0%	2.0%
female	18-34	5.6%	18.1%	14.3%	12.7%	14.3%	22.3%
female	35-54	16.0%	16.9%	0.0%	25.6%	31.6%	22.2%
female	55-74	20.8%	16.0%	14.3%	17.7%	15.8%	10.0%
female	75+	18.4%	5.6%	0.0%	2.1%	3.0%	3.3%
total	18+	100%	100%	100%	100%	100%	100%

Table A2. OpenBUGS parameter estimates, by country

	United Kingdom	Japan	United States
beta[0]	0.91 (0.90 — 0.92) ***	0.85 (0.84 — 0.86) ***	0.88 (0.87 — 0.89) ***
beta[1]	-0.06 (-0.07 — -0.06) **	-0.10 (-0.11 — -0.08) **	-0.04 (-0.04 — -0.03) **
beta[2]	-0.35 (-0.37 — -0.34) **	-0.43 (-0.46 — -0.40) **	-0.33 (-0.35 — -0.31) **
beta[3]	-0.13 (-0.13 — -0.12) **	-0.05 (-0.07 — -0.04) **	-0.08 (-0.09 — -0.07) **
beta[4]	-0.26 (-0.28 — -0.25) **	-0.09 (-0.12 — -0.07) **	-0.25 (-0.27 — -0.24) **
beta[5]	-0.08 (-0.09 — -0.08) **	-0.03 (-0.05 — -0.02) **	-0.05 (-0.05 — -0.04) **
beta[6]	-0.23 (-0.24 — -0.21) **	-0.12 (-0.15 — -0.10) **	-0.19 (-0.20 — -0.17) **
beta[7]	-0.09 (-0.10 — -0.08) **	-0.06 (-0.07 — -0.04) **	-0.05 (-0.06 — -0.04) **
beta[8]	-0.47 (-0.48 — -0.46) **	-0.17 (-0.19 — -0.15) **	-0.36 (-0.37 — -0.34) **
beta[9]	-0.12 (-0.13 — -0.12) **	-0.06 (-0.07 — -0.05) **	-0.08 (-0.09 — -0.08) **
beta[10]	-0.38 (-0.40 — -0.37) **	-0.11 (-0.13 — -0.10) **	-0.28 (-0.30 — -0.27) **
gamma[1]	-0.50 (-0.51 — -0.49) **	-0.69 (-0.73 — -0.66) **	-0.51 (-0.53 — -0.50) **
gamma[2]	0.41 (0.37 — 0.45) **	-0.39 (-0.62 — -0.13) **	0.46 (0.41 — 0.52) **
gamma[3]	-0.86 (-0.95 — -0.76) **	0.16 (-0.94 — 1.70)	-0.56 (-0.69 — -0.44) **
gamma[4]	-1.12 (-1.19 — -1.06) **	-1.02 (-4.32 — 1.77)	-1.26 (-1.42 — -1.10) **
gamma[5]	-0.06 (-0.17 — 0.06)	-0.34 (-2.39 — 1.72)	-0.03 (-0.27 — 0.22)
scale_MO[1,1]	1.08 (0.98 — 1.18)	0.96 (0.82 — 1.10)	1.02 (0.92 — 1.12)
scale_MO[1,2]	1.01 (0.91 — 1.11)	1.00 (0.88 — 1.12)	1.10 (1.00 — 1.21)
scale_MO[1,3]	0.95 (0.85 — 1.05)	0.98 (0.86 — 1.10)	1.07 (0.94 — 1.21)
scale_MO[1,4]	1.41 (1.23 — 1.58)	0.91 (0.65 — 1.19)	0.90 (0.68 — 1.12)
scale_MO[2,1]	0.91 (0.82 — 1.00) ***	1.02 (0.89 — 1.15)	1.00 (0.91 — 1.09)
scale_MO[2,2]	0.94 (0.85 — 1.03)	1.07 (0.96 — 1.18)	1.00 (0.92 — 1.10)
scale_MO[2,3]	0.82 (0.73 — 0.91) ***	1.04 (0.93 — 1.16)	0.98 (0.87 — 1.10)
scale_MO[2,4]	0.89 (0.75 — 1.02)	1.03 (0.79 — 1.29)	0.92 (0.74 — 1.10)
scale_SC[1,1]	0.81 (0.71 — 0.90) ***	0.88 (0.51 — 1.30)	1.05 (0.93 — 1.18)
scale_SC[1,2]	0.67 (0.58 — 0.77) ***	0.79 (0.48 — 1.16)	0.78 (0.67 — 0.88) ***
scale_SC[1,3]	1.01 (0.90 — 1.12)	1.75 (1.32 — 2.25)	0.88 (0.72 — 1.05)
scale_SC[1,4]	1.26 (1.09 — 1.44) ***	0.92 (0.46 — 1.55)	0.90 (0.63 — 1.17)
scale_SC[2,1]	0.69 (0.61 — 0.78) ***	0.76 (0.45 — 1.13)	1.00 (0.90 — 1.10)
scale_SC[2,2]	0.74 (0.65 — 0.84) ***	1.04 (0.72 — 1.39)	0.84 (0.74 — 0.94) ***
scale_SC[2,3]	1.26 (1.16 — 1.37) ***	1.08 (0.72 — 1.48)	0.98 (0.85 — 1.13)
scale_SC[2,4]	1.55 (1.41 — 1.70) ***	0.77 (0.38 — 1.30)	1.57 (1.37 — 1.77) ***
scale_UA[1,1]	0.98 (0.87 — 1.10)	0.82 (0.53 — 1.13)	0.84 (0.70 — 0.98) ***
scale_UA[1,2]	0.87 (0.75 — 1.00) ***	0.88 (0.62 — 1.16)	0.88 (0.74 — 1.02)
scale_UA[1,3]	1.05 (0.94 — 1.18)	1.12 (0.83 — 1.44)	1.06 (0.85 — 1.27)
scale_UA[1,4]	0.92 (0.71 — 1.12)	0.87 (0.45 — 1.43)	1.21 (0.85 — 1.55)
scale_UA[2,1]	0.85 (0.74 — 0.96) ***	1.28 (0.99 — 1.62)	0.87 (0.75 — 1.00) ***
scale_UA[2,2]	0.90 (0.79 — 1.01)	1.10 (0.86 — 1.37)	0.78 (0.67 — 0.90) ***
scale_UA[2,3]	1.10 (0.98 — 1.22)	1.11 (0.83 — 1.40)	1.15 (0.98 — 1.34)
scale_UA[2,4]	1.33 (1.17 — 1.49) ***	0.81 (0.44 — 1.28)	1.22 (0.98 — 1.47)

Table A2 (continued)

scale_PD[1,1]	0.92 (0.86 — 0.98) ***	1.23 (1.00 — 1.46) ***	0.95 (0.88 — 1.03)
scale_PD[1,2]	0.91 (0.84 — 0.97) ***	0.92 (0.75 — 1.10)	0.98 (0.90 — 1.05)
scale_PD[1,3]	0.94 (0.88 — 1.00) ***	1.05 (0.87 — 1.25)	0.94 (0.83 — 1.04)
scale_PD[1,4]	1.07 (0.96 — 1.18)	0.48 (0.26 — 0.79) ***	0.76 (0.59 — 0.93) ***
scale_PD[2,1]	1.11 (1.05 — 1.17) ***	1.14 (0.95 — 1.35)	1.02 (0.95 — 1.09)
scale_PD[2,2]	1.03 (0.97 — 1.09)	1.11 (0.95 — 1.28)	1.09 (1.02 — 1.16) ***
scale_PD[2,3]	0.99 (0.93 — 1.04)	1.32 (1.13 — 1.52) ***	1.02 (0.94 — 1.11)
scale_PD[2,4]	1.04 (0.96 — 1.13)	0.75 (0.45 — 1.12)	1.24 (1.11 — 1.38) ***
scale_AD[1,1]	0.80 (0.73 — 0.86) ***	1.06 (0.78 — 1.35)	0.85 (0.77 — 0.94) ***
scale_AD[1,2]	0.84 (0.77 — 0.90) ***	0.99 (0.76 — 1.23)	0.81 (0.73 — 0.89) ***
scale_AD[1,3]	0.83 (0.77 — 0.90) ***	1.39 (1.14 — 1.66) ***	1.03 (0.91 — 1.15)
scale_AD[1,4]	1.22 (1.10 — 1.34) ***	0.54 (0.29 — 0.89) ***	1.28 (1.08 — 1.48) ***
scale_AD[2,1]	1.03 (0.97 — 1.10)	0.85 (0.61 — 1.09)	0.88 (0.80 — 0.95) ***
scale_AD[2,2]	0.99 (0.93 — 1.06)	1.01 (0.81 — 1.23)	0.95 (0.88 — 1.03)
scale_AD[2,3]	1.10 (1.04 — 1.17) ***	1.25 (1.02 — 1.49) ***	1.05 (0.95 — 1.15)
scale_AD[2,4]	1.20 (1.10 — 1.29) ***	0.91 (0.52 — 1.37)	1.15 (0.99 — 1.32)

** Mean posterior estimates with 95% credible intervals (CI) in parenthesis*

*** 95% CI does not contain 0 *** 95% CI does not contain 1*

Table A3. US survey respondents and US nationally representative benchmark, by race/ethnicity, age group, and year*

		United States			
		dataset	national population		
race/ethnicity	Age	(N=3,915)	2004	2022	Δ
white (non-Hispanic)	18-34	12.1%	21.6%	17.9%	-3.7%
white (non-Hispanic)	35-54	16.0%	30.3%	20.7%	-9.6%
white (non-Hispanic)	55-74	9.0%	18.1%	22.2%	4.1%
white (non-Hispanic)	75+	3.4%	6.5%	7.7%	1.2%
black/African American	18-34	10.8%	3.9%	4.3%	-0.4%
black/African American	35-54	11.6%	4.6%	4.3%	-0.3%
black/African American	55-74	4.7%	2.0%	3.4%	1.4%
black/African American	75+	1.1%	0.6%	0.8%	0.2%
Hispanic/Latino	18-34	15.4%	5.7%	7.1%	1.4%
hispanic/Latino	35-54	11.2%	4.7%	7.0%	2.3%
Hispanic/Latino	55-74	3.8%	1.6%	3.7%	2.1%
hispanic/Latino	75+	0.7%	0.4%	0.8%	0.4%
total	18+	100%	100%	100%	

** Note: US national population estimates obtained from the American Community Survey of the United States Census Bureau (https://www.census.gov/programs-surveys/acs)*

Table A4. US EQ-5D health state decrements, original and ethnicity/age corrected

	United States		
	2002	2022	Δ
MO2	-0.04<br>(-0.05 — -0.03)	-0.04<br>(-0.05 — -0.03)	0.00<br>(0.00 — 0.00)
MO3	-0.35<br>(-0.37 — -0.33)	-0.34<br>(-0.36 — -0.33)	0.00<br>(-0.01 — 0.00)
SC2	-0.07<br>(-0.08 — -0.06)	-0.07<br>(-0.08 — -0.06)	0.00<br>(0.00 — 0.00)
SC3	-0.22<br>(-0.24 — -0.2)	-0.22<br>(-0.24 — -0.21)	0.00<br>(0.00 — 0.01)
UA2	-0.05<br>(-0.06 — -0.04)	-0.05<br>(-0.06 — -0.05)	0.00<br>(0.00 — 0.00)
UA3	-0.16<br>(-0.18 — -0.15)	-0.16<br>(-0.18 — -0.15)	0.01<br>(0.00 — 0.01)
PD2	-0.06<br>(-0.06 — -0.05)	-0.06<br>(-0.06 — -0.05)	0.00<br>(0.00 — 0.00)
PD3	-0.38<br>(-0.4 — -0.37)	-0.38<br>(-0.4 — -0.37)	0.00<br>(0.00 — 0.00)
AD2	-0.08<br>(-0.09 — -0.08)	-0.08<br>(-0.09 — -0.08)	0.00<br>(0.00 — 0.00)
AD3	-0.28<br>(-0.29 — -0.26)	-0.28<br>(-0.29 — -0.26)	0.00<br>(0.00 — 0.00)

Table A5. OpenBUGS parameter estimates with ethnicity/age scale factors

	United States
beta[0]	0.89 (0.88 — 0.89) ***
beta[1]	-0.04 (-0.04 — -0.03) **
beta[2]	-0.32 (-0.34 — -0.3) **
beta[3]	-0.08 (-0.09 — -0.07) **
beta[4]	-0.25 (-0.27 — -0.23) **
beta[5]	-0.06 (-0.06 — -0.05) **
beta[6]	-0.18 (-0.19 — -0.16) **
beta[7]	-0.05 (-0.06 — -0.04) **
beta[8]	-0.34 (-0.36 — -0.33) **
beta[9]	-0.08 (-0.09 — -0.07) **
beta[10]	-0.27 (-0.29 — -0.26) **
gamma[1]	-0.52 (-0.54 — -0.51) **
gamma[2]	0.45 (0.40 — 0.50) **
gamma[3]	-0.41 (-0.51 — -0.31) **
gamma[4]	-1.13 (-1.26 — -0.97) **
gamma[5]	-0.40 (-0.59 — -0.22) **
scale_MO[1,1]	1.18 (1.04 — 1.32) ***
scale_MO[1,2]	1.10 (0.99 — 1.22)
scale_MO[1,3]	1.21 (1.09 — 1.35) ***
scale_MO[1,4]	0.76 (0.56 — 0.97) ***
scale_MO[2,1]	0.91 (0.78 — 1.04)
scale_MO[2,2]	0.93 (0.82 — 1.04)
scale_MO[2,3]	1.06 (0.88 — 1.24)
scale_MO[2,4]	1.05 (0.78 — 1.34)
scale_MO[3,1]	1.00 (0.88 — 1.11)
scale_MO[3,2]	1.20 (1.08 — 1.33) ***
scale_MO[3,3]	0.81 (0.64 — 0.99)
scale_MO[3,4]	0.80 (0.48 — 1.16) ***
scale_SC[1,1]	0.83 (0.70 — 0.97) ***
scale_SC[1,2]	0.81 (0.69 — 0.93) ***
scale_SC[1,3]	0.74 (0.60 — 0.89) ***
scale_SC[1,4]	1.56 (1.32 — 1.80) ***
scale_SC[2,1]	0.96 (0.84 — 1.10)
scale_SC[2,2]	0.91 (0.79 — 1.02)
scale_SC[2,3]	0.88 (0.71 — 1.05)
scale_SC[2,4]	1.05 (0.72 — 1.39)
scale_SC[3,1]	1.25 (1.12 — 1.38) ***
scale_SC[3,2]	0.78 (0.66 — 0.91) ***
scale_SC[3,3]	1.30 (1.08 — 1.50) ***
scale_SC[3,4]	0.92 (0.61 — 1.26)

Table A5 (continued)

scale_UA[1,1]	0.89 (0.72 — 1.06)
scale_UA[1,2]	0.85 (0.72 — 1.00)
scale_UA[1,3]	1.29 (1.08 — 1.52) ***
scale_UA[1,4]	1.63 (1.32 — 1.96) ***
scale_UA[2,1]	0.51 (0.37 — 0.65) ***
scale_UA[2,2]	0.51 (0.39 — 0.64) ***
scale_UA[2,3]	0.79 (0.58 — 1.01)
scale_UA[2,4]	0.74 (0.39 — 1.13)
scale_UA[3,1]	1.15 (1.00 — 1.32)
scale_UA[3,2]	1.15 (0.99 — 1.33)
scale_UA[3,3]	1.38 (1.12 — 1.66) ***
scale_UA[3,4]	1.13 (0.67 — 1.63)
scale_PD[1,1]	0.98 (0.89 — 1.07)
scale_PD[1,2]	1.18 (1.09 — 1.27) ***
scale_PD[1,3]	1.14 (1.04 — 1.25) ***
scale_PD[1,4]	1.40 (1.26 — 1.57) ***
scale_PD[2,1]	1.23 (1.14 — 1.32) ***
scale_PD[2,2]	1.06 (0.96 — 1.15)
scale_PD[2,3]	0.96 (0.84 — 1.10)
scale_PD[2,4]	0.53 (0.34 — 0.72) ***
scale_PD[3,1]	0.99 (0.91 — 1.07)
scale_PD[3,2]	1.06 (0.97 — 1.15)
scale_PD[3,3]	0.93 (0.78 — 1.08)
scale_PD[3,4]	0.53 (0.33 — 0.81) ***
scale_AD[1,1]	0.98 (0.88 — 1.09)
scale_AD[1,2]	1.04 (0.95 — 1.13)
scale_AD[1,3]	1.10 (0.99 — 1.21)
scale_AD[1,4]	1.23 (1.05 — 1.43) ***
scale_AD[2,1]	0.80 (0.71 — 0.91) ***
scale_AD[2,2]	0.79 (0.69 — 0.89) ***
scale_AD[2,3]	1.08 (0.93 — 1.24)
scale_AD[2,4]	1.24 (1.01 — 1.49) ***
scale_AD[3,1]	0.89 (0.80 — 0.99) ***
scale_AD[3,2]	0.96 (0.86 — 1.06)
scale_AD[3,3]	1.00 (0.83 — 1.17)
scale_AD[3,4]	0.90 (0.64 — 1.20)

** Mean posterior estimates with 95% credible intervals (CI) in parenthesis*

*** 95% CI does not contain 0 *** 95% CI does not contain 1*
